# Assessment of the Risk to Public Health due to Use of Antimicrobials in Pigs—An Example of Pleuromutilins in Denmark

**DOI:** 10.3389/fvets.2017.00074

**Published:** 2017-05-26

**Authors:** Lis Alban, Johanne Ellis-Iversen, Margit Andreasen, Jan Dahl, Ute W. Sönksen

**Affiliations:** ^1^Risk Assessment Group, Department for Food Safety and Veterinary Issues, Danish Agriculture and Food Council, Copenhagen, Denmark; ^2^National Food Institute, Technical University of Denmark, Kongens Lyngby, Denmark; ^3^Danish Association of the Veterinary Pharmaceutical Industry, Copenhagen, Denmark; ^4^Department for Bacteria, Parasites and Fungi, Statens Serum Institut, Copenhagen, Denmark

**Keywords:** pleuromutilins, antimicrobial resistance, treatment guidelines, risk assessment, risk management, pigs, one health, human health

## Abstract

Antibiotic consumption in pigs can be optimized by developing treatment guidelines, which encourage veterinarians to use effective drugs with low probability of developing resistance of importance for human health. In Denmark, treatment guidelines for use in swine production are currently under review at the Danish Veterinary and Food Administration. Use of pleuromutilins in swine has previously been associated with a very low risk for human health. However, recent international data and sporadic findings of novel resistance genes suggest a change of risk. Consequently, a reassessment was undertaken inspired by a risk assessment framework developed by the European Medicines Agency. Livestock-associated methicillin-resistant *Staphylococcus aureus* of clonal complex 398 (MRSA CC398) and enterococci were identified as relevant hazards. The release assessment showed that the probability of development of pleuromutilin resistance was high in MRSA CC398 (medium uncertainty) and low in enterococci (high uncertainty). A relatively small proportion of Danes has an occupational exposure to pigs, and foodborne transmission was only considered of relevance for enterococci, resulting in an altogether low exposure risk. The human consequences of infection with pleuromutilin-resistant MRSA CC398 or enterococci were assessed as low for the public in general but high for vulnerable groups such as hospitalized and immunocompromised persons. For MRSA CC398, the total risk was estimated as low (low uncertainty), among other due to the current guidelines on prevention of MRSA in place at Danish hospitals, which include screening of patients with daily contact to pigs on admittance. Moreover, MRSA CC398 has a medium human–human transmission potential. For enterococci, the total risk was estimated as low due to low prevalence of resistance, low probability of spread to humans, low virulence, but no screening of hospitalized patients, high ability of acquiring resistance genes, and a limited number of alternative antimicrobials (high uncertainty). This assessment reflects the current situation and should be repeated if pleuromutilin consumption increases substantially, resulting in increased prevalence of mobile, easily transmissible resistance mechanisms. Continuous monitoring of pleuromutilin resistance in selected human pathogens should therefore be considered. This also includes monitoring of linezolid resistance, since resistance mechanisms for pleuromutilins and oxazolidones are often coupled.

## Introduction

In Denmark, all antimicrobials used for animals are prescribed by veterinary practitioners and sold through pharmacies or feed mills. Since year 2000, all antimicrobial prescription and sales records have been collated in a national database called VetStat. A prescription record includes information about type, concentration and amount of the antimicrobial used, animals treated, indication, age group, herd identification, name of prescriber, and livestock producer (1).

Since 1995, antimicrobial consumption and antimicrobial resistance in bacteria from animals, food, and humans have been monitored routinely as part of a coordinated national program: Danish Integrated Antimicrobial Resistance Monitoring and Research program (DANMAP) (2). The monitoring of antimicrobial resistance covers three categories of bacteria: human and animal pathogens, zoonotic bacteria, and indicator bacteria. Each year, a DANMAP report summarizes the results of the monitoring program and related research, all available in: www.danmap.org. The program is run by a One Health consortium of Statens Serum Institut, the National Veterinary Institute, and the National Food Institute.

The continuous surveillance has increased awareness of the relationship between antimicrobial consumption in animals and development of resistance. Several initiatives exist to control and reduce the consumption in pigs. These include the Specific Pathogen Free (SPF) health scheme, banning of growth promoters, prohibition of prophylactic usage, and a control program called the “Yellow Card scheme,” introduced in 2010. This administrative system defines limits to antimicrobial consumption per age group within a swine herd per time period (3). These limits have further been reduced and enforced as the national average of consumption decreased (4). Recently, a weighting of different antimicrobial classes according to their potential for generating antimicrobial resistance was introduced in the scheme to steer prescribers toward certain antimicrobial choices (5).

In addition, a working group consisting of stakeholder representatives and headed by the Danish Veterinary and Food Administration developed the first generation of antimicrobial treatment guidelines for pigs in 2006. The guidelines help the veterinarian in choosing the most appropriate drug for the respective infectious disease encountered. In the most recent version from 2010, the guidelines contain specific recommendations on the choice of drug as well as background information on the known percentage of resistance in the suspected causal pathogen, pharmacodynamics of the drug, and the probability of development of antimicrobial resistance transmissible to humans.

The first Danish guidelines were developed without formal risk assessments, and this led to some controversy regarding the assessment of macrolides, resulting in a more thorough estimation of this risk (6). In the newest version, a simplified risk assessment approach developed by the US authorities (7) was used, resulting in a broader acceptance of the guidelines by the stakeholders. The possible impact on human health regarding each antimicrobial class was assessed using a scale with four levels: very high, high, medium, and low. Third and fourth generations’ cephalosporins and fluoroquinolones were considered to be of very high risk for the development of ESBL-producing or fluoroquinolone-resistant bacteria and for transmission to humans of these, therefore, these antimicrobial classes were recommended not to be used. Ampicillin/amoxicillin, gentamycin/apramycin, tylosin/lincomycin, and tetracyclines were assessed to have a high risk, and their use should therefore be limited. Florfenicol, penicillin, tulathromycin/tilmicosin, and sulfonamide/trimethroprim were assessed as medium risk, and neomycin, colistin, and pleuromutilins as low or very low risk (7).

Currently, the guidelines are being revised. The part of the guidelines that cover the assessment on the risk to human health is built on a risk assessment framework inspired by a set of guidelines issued by the European Medicines Agency (EMA) in February 2015 (8). Currently, these guidelines exist only in a drafted version.

In this article, the risk assessment for pleuromutilins will be presented. Pleuromutilins consist of a class of antimicrobials, of which tiamulin and valnemulin are of veterinary use exclusively and primarily used to treat diseases in swine. Pleuromutilins have so far been associated with a low probability of development of antimicrobial resistance of importance for human health, since no equivalent drug exists for human treatment and no important cross-resistances to other human antimicrobials have been described. In the 2010 version of the Danish treatment guidelines, tiamulin and valnemulin were therefore recommended for treatment of relevant infections in swine (see Hazard identification section for a specification).

However, it was decided to reassess the risk related to the use of pleuromutilins in Danish swine due to:

1. New knowledge on resistance to pleuromutilins emerging on mobile elements in staphylococci and enterococci among other, making horizontal transmission and coselection of other critical resistances such as linezolid resistance possible.
2. The potential selection of pleuromutilin-resistant staphylococci [including livestock-associated methicillin-resistant *Staphylococcus aureus* (MRSA) of clonal complex 398, MRSA CC398] in swine treated with pleuromutilins indicated by international literature (9).
3. Possible increased importance of pleuromutilins for the treatment of humans due to new product developments (9).
4. Expectations on an increase in the use of pleuromutilins in the Danish swine production due to changes in the Danish Yellow Card scheme using weighting of the different antimicrobial classes.

The objective of this article is to assess the risk for human health associated with the use of pleuromutilins in the Danish swine production.

## Materials and Methods

### Materials

The risk assessment used several sources of information, the primary source being Danish data obtained through Danish surveillance programs and research. For each aspect, the newest data available were obtained. Data describing the consumption of pleuromutilins in Danish livestock in 2015 were obtained from VetStat. The Danish pleuromutilin consumption was compared with that of other European countries using data from ESVAC 2010 reported by EMA (9). Data from DANMAP 2015 were obtained to describe the prevalence of resistance observed in Denmark (2). The Danish-resistance data were compared to EU data from 2013 (10). A central source was the EMA’s reflection paper from 2013, regarding the use of pleuromutilins in food-producing animals in the European Union (9). Finally, various scientific publications were used for scientific information required to assess the risk for humans related to the use of pleuromutilins in Danish swine.

### Methods

**Figure 1:**
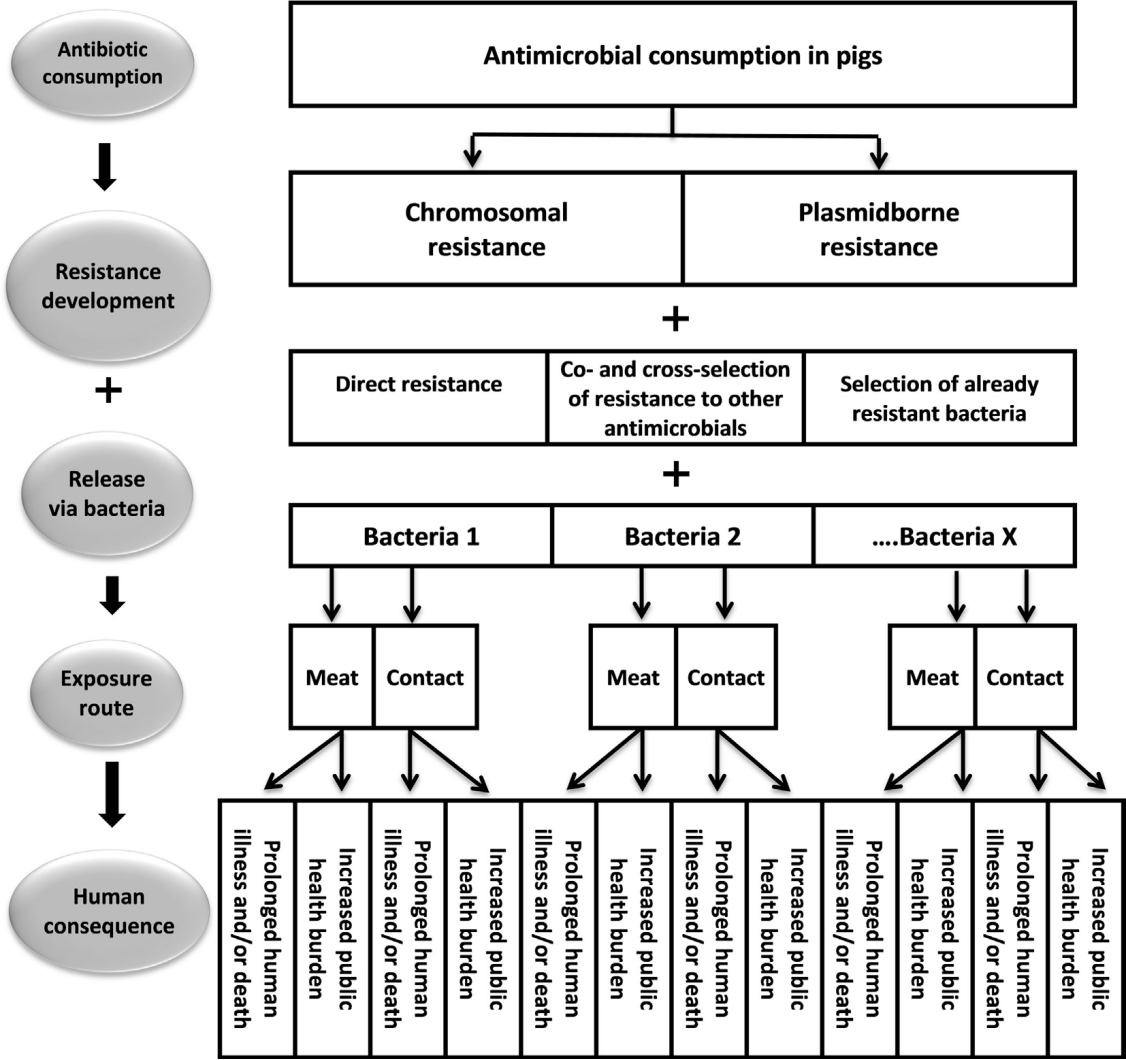
**A schematic framework illustrating the risk pathway from consumption of pleuromutilin in pigs to the risk to public health**.

We applied an adapted version of EMA’s framework on assessment of risk to public health from antimicrobial resistance due to the use of an antimicrobial veterinary product in food-producing animals (8). In essence, it follows the International Organization for Animal Health’s (OIE) approach to risk assessment and consists of four steps describing the risk pathway combined into a risk estimate (Figure 1).

The first step was the hazard identification. This step identified antimicrobial-resistant bacteria or resistance determinants that could result in human illness and, hence, may be selected due to the use of pleuromutilins in Danish swine. Hazards for further analysis were identified by literature and expert opinion, and a preliminary assessment through the four steps of the risk pathway was carried out (Figure 1). If the hazard had potential to fulfill all steps in the risk pathway, it was included in the subsequent risk assessment. Lack of information in any important step also excluded potential hazard from further analysis.

The second step was the release assessment. In this step, the presence and multitude of the identified hazards in the swine reservoir as a result of the use of pleuromutilins in Danish swine were described and assessed.

The third step was the exposure assessment, which addressed the biological pathways necessary for exposure of humans to the identified hazards, which are the selected resistances from the release from swine. The exposure assessment addressed the two main pathways from pigs to humans: meat and direct contact. An exposure was defined as a contact that resulted in the person becoming either carrier or infected by the hazard. Each route was considered separately and combined into one exposure assessment score per hazard for the Danish situation.

A scale was used to describe the probability of release and exposure: very low (<2%), low (2–10%), medium (11–30%), and high (>30%).

The fourth step was the consequence assessment. The consequences of infection by resistant hazards caused by pleuromutilin consumption in pigs were assessed by two effects: (1) an increase in public health burden or (2) prolonged human illness and/or death. The public health burden was defined as a marked increase in the total number of carriers or infected people. This could pose a risk to society due to increased expenses related to the control of spread (screening and isolation procedures of patients), loss of work days, and an overall higher risk of severe illness due to increased exposure. Prolonged illness and/or death were measures for the severity of treatment failure in the individual patient due to resistance caused by pleuromutilin usage in pigs. Each consequence was assessed on a scale of very low, low, medium, or high based on the increase in cases caused by the resistance and the importance of the pleuromutilin and related antibiotics in treatment in Denmark.

The risk estimate was a summary of the steps above, and it was based on a combination of the probability of the unwanted event and the associated consequences. If any of the probabilities related to release, exposure, or consequences were zero, there would be no risk.

The level of uncertainty was described as high, medium, or low. Low uncertainty was used, where abundant data pointing in one direction were available, medium where only a limited amount of data were available, and high where no data were available, and expert opinion had to be used instead.

A detailed description of what is included in the different elements is described by EMA (8), and a qualitative assessment is recommended in the presence of substantial data gaps. Any deviation due to lack of data has been indicated.

The EMA framework is very detailed and thereby contains many elements, reflecting its purpose as a tool of risk assessment in regard to application for marketing authorizations for new antimicrobial classes (8). Pleuromutilins have been used for veterinary purposes for decades, and therefore, direct data are available describing consumption, resistance, transmission, and consequences. Some of the listed elements were therefore found to be of no relevance for the present exercise and were therefore excluded from this article.

## Results

### Hazard Identification

#### Pleuromutilin Consumption in Denmark

Pleuromutilins for treatment of swine entered the Danish market in the early 1980s. The antimicrobial class pleuromutilins includes tiamulin and valnemulin, which are semisynthetic derivatives (11, 12). In Denmark, they are registered for indications including porcine proliferative enteropathy (due to *Lawsonia intracellularis*), swine dysentery (due to *Brachyspira hyodysenteriae*), porcine colonic spirochetosis (due to *Brachyspira pilosicoli*), enzootic pneumonia (due to *Mycoplasma hyopneumoniae*), infections caused by *Mycoplasma hyorhinis* and *M. hyosynoviae*, and respiratory diseases caused by tiamulin-sensitive microorganisms (13). They can be administered either by injection or added to feed or water, allowing both individual and group treatment (14).

Almost all pleuromutilins (99.9%) prescribed for Danish livestock are used in swine production, and the main area of use is group treatments, which is usually short term. In 2015, the annual consumption measured in active kilogram compound was 7,878 kg (2). This is close to the lowest consumption measured in 2011 and 2012, following the implementations of the yellow card scheme and the new revised version of the treatment guidelines (15). In 2015, the consumption corresponded to 10% of the total use of antimicrobials in swine in Denmark (2). Based on the consumption in kilogram, half of the amount (51%) was used in finishing pigs, 39% in weaner pigs, and 10% in sows and piglets. When measured in the daily dose per 1,000 animals (DAPD), the consumption has varied from 3 to 6 DAPD during the past decade, also here the fluctuations observed mirror the mentioned effect of the initiatives on prudent use and reduction (2). In 2015, the consumption was 4.4 DAPD.

Between the European countries, a large variation in consumption of pleuromutilins is observed. In 2010, the top five countries were Portugal, Hungary, Spain, Czech Republic, and the United Kingdom, whereas Denmark figured toward the lower end (9).

For the treatment of humans, only one pleuromutilin-containing product, called Retapamulin, was previously registered on the Danish market but was withdrawn in 2014, probably due to low sales. Retapamulin was licensed for topical use for the treatment of impetigo and infected small lacerations, abrasions, or sutured wounds.

A new pleuromutilin, called Lefamulin, is currently under development and entered its second phase III clinical trial in April 2016 (16). Lefamulin is developed to be the first pleuromutilin for systemic use in humans. In microbiological studies, Lefamulin showed potent *in vitro* activity against *S. pneumonia*e, *H. influenzae, M. pneumoniae, L. pneumophila*, and *C. pneumoniae*, as well as *S. aureus*, including multidrug resistant strains of *S. pneumonia* and MRSA. In Denmark, only the latter seems relevant for future treatment with Lefamulin, since the occurrence of drug resistance in the other target bacteria is very low and hardly any multidrug resistance is found in these (2). This drug is expected to be registered for the treatment of serious pneumonia and may also find its use in the treatment of rare cases of genital tract infections with multidrug-resistant *Ureaplasma*. The US authorities FDA have granted fast track designation to the oral formulation of Lefamulin to facilitate the development of a drug that is able to treat serious conditions and fill an unmet medical need (16). This increases the need to focus on how to limit the probability of developing pleuromutilin resistance and to monitor resistance development.

Pleuromutilins are closely related to oxazolidones due to very similar chemical structures and coresistance might thus occur. In Denmark, two oxazolidones are registered for human treatment: Tedizolidphosphat and linezolid. Tedizolidphosphat is only registered for specific and complicated skin structure infections, and the consumption is negligible. Linezolid is registered for the treatment of severe infections with gram-positives. It is recommended for complicated infections only and where other antimicrobials cannot be used, either due to allergies or due to development of resistance. The consumption of linezolid is only minor compared to other antimicrobial classes and currently accounts for less than 0.5% of the total consumption at hospitals and close to 0% in primary health care in Denmark (2). Treatment periods of more than 28 days are not recommended due to the risk of adverse effects. To date, linezolid is primarily used in hospitals for complicated staphylococcal bone infections, for the treatment of complicated infections with MRSA, and for the treatment of severe infections with penicillin-resistant pneumococci in Denmark. It is also registered for the treatment of infectious endocarditis caused by vancomycin-resistant enterococci (VRE), but very rarely used, since this is an extremely rarely occurring condition. It is, thus, considered a last line drug.

#### Known Resistance Determinants

Usage of pleuromutilins in pigs may influence public health in the following ways (Figure 1):

1. Induce and select chromosomal resistance by point mutations in the 23S rRNA and *rplC* genes and thereby
  i.exhibit cross-resistance to linezolid.
2. Induce and select plasmid-borne resistance via transferable resistance genes *cfr* and *vga* and thereby
  i.exhibit cross- and coselection of resistance to or from other antibiotic groups.
3. Select for already pleuromutilin-resistant bacteria.

Emergence of resistance due to chromosomal point mutations is usually not considered critical due to a limited capacity to spread, unless the resistant clone has a high pathogenicity or its ability to spread between humans for some reason is high. However, some of the pleuromutilin point mutations in *Staphylococcus* spp. have been found to lead to cross-resistance with linezolid (17, 18). Point mutation-driven resistance to pleuromutilin has also been reported to gradually reduce susceptibility in *Brachyspirae* in pigs, making continued use in swine herds of some concern due to the risk of treatment failure in these (19–21). This has no direct effect on human health since *Brachyspirae* only very rarely cause human disease, but similar mechanisms in human bacteria may be of concern for human treatment if the drug is to be used extensively in humans in the future.

Pleuromutilin resistance may also develop as or be acquired by horizontal transfer of mobile genetic elements such as plasmids or transposons containing resistance genes and might spread not only within but also between bacterial species. Such elements are more likely to spread at a high speed among bacterial populations than resistance driven by point mutations and may affect the susceptibility of important human pathogens rapidly. Among these there are several gene families of relevance to pleuromutilin usage: the methyltransferase genes *cfr* and the *vga* ABC transporter genes (22–25).

*cfr* genes can confer multidrug resistance and have great potential to spread. They have been found in staphylococci of pig origin including MRSA CC398 (26, 27). *cfr* genes can confer cross-resistance between pleuromutilins, lincosamides, streptogramin A, phenicols, and oxazolidinones (=linezolid) (9, 25). However, a national prevalence survey of MRSA CC398 in pigs in Denmark in 2014 did not reveal any *cfr* genes in the 275 MRSA CC398 isolates (Personal communication, J. Larsen, Statens Serum Institut, 2017). A *cfr*-like gene conferring phenotypic resistance with high MIC for especially clindamycin and linezolid was reported in different *Bacillales* and in a Danish strain of *Clostridium difficile*. It is yet to be established whether this has any clinical importance in humans in Denmark (28). *cfr* genes have also been reported in enterococci from animal origin in China (29). No further reports on a significant spread of *cfr* genes in enterococci were found from other countries, but the occurrence of *cfr* genes in enterococci is worrying since enterococci have high ability to share plasmids.

The ABC transporters induce cross-resistance between pleuromutilins, lincosamides, and streptogramin A and have the potential to cross-select for further antimicrobial classes (30, 31). The possibility for coresistance to other antimicrobial classes will depend on the mobile genetic elements circulating in the bacterial population. Plasmids carrying *vga*(C) genes have been found to contain the tetracycline-resistance gene *tet*(L), the kanamycin/neomycin-resistance gene *aadD*, and the trimethoprim-resistance gene *dfrK* (32). Staphylococci carrying *vga*(E) genes were also found resistant to beta-lactams, tetracyclines, trimethoprim, macrolides, lincosamides, and spectinomycin and resistant to or with reduced susceptibility to quinupristin/dalfopristin (33). The 275 MRSA CC398 from the pig survey in Denmark in 2014 revealed only one isolate carrying a *vga* gene (Personal communication, J. Larsen, Statens Serum Institut, 2017).

Other mobile-resistance elements such as *optrA* genes have been reported in pork in other countries, but in Denmark, the *optrA* gene has to date only been reported in three enterococcal isolates from imported turkey, one isolate from Danish calf, and one human clinical *Enterococcus faecalis* isolate (34). They confer resistance to phenicols and the newest generation of oxazolidinones including linezolid (35–38). *optrA* genes do not confer resistance to pleuromutilines but might be carried together with *cfr* genes on the same plasmid (37). Thus, the coselection with pleuromutilins resistance may result in wider spread, if the consumption of pleuromutilins increases.

The use of pleuromutilins may thus excerpt a selective pressure for multidrug resistant staphylococci, enterococci, and other gram-positives, if the level of pleuromutilin resistance increases.

Hence, the main risk of pleuromutilin usage in pigs is multiresistance transfer and development of coresistance to linezolid, which is a last line antibiotic in humans.

#### Potential Bacterial Species of Importance for Human Health

Pleuromutilin resistance is not routinely monitored in Denmark (2). Theoretically, resistance may develop in all treatment targeted pathogens: *Brachyspirae, Mycoplasma* spp., and *Lawsonia* and other pig pathogens such as *Actinobacillus, Erysipelothrix*, and *Pasteurella*. Data from the Danish swine industry’s laboratory in Kjellerup show that all 210 tested *A. pleuropneumoniae* isolates from diseased pigs were sensitive to pleuromutilins. No information regarding *B. hyodysenteria* was available at the laboratory due to dysentery being very uncommon in Danish pig production (Personal comment, B. Svensmark, SEGES, 2017). A Swedish study testing clinical isolates of *Brachyspira hyodysenteriae* and *B. pilosicoli* showed slowly increasing resistances over a 20-year period (from 1990 till 2010) and declining resistances for the last 2–3 years of the study (19). Recent Swedish surveillance data have revealed that 94% of 69 Swedish *B. hyodysenteria* isolates were sensitive to tiamulin (39). Hence, the level of resistance in these microorganisms is considered to be low. Moreover, these organisms very rarely cause disease in humans. They were therefore not considered relevant for this risk assessment.

Anaerobe species such as *Clostridium* spp. and specifically *Clostridium difficile* were considered to be of potential relevance, as they can spread from pigs to humans. As mentioned previously, observation of *cfr* genes and pleuromutilin resistance in a strain of *C. difficile* has been reported (28), but the involved isolate was not associated with resistance to metronidazole, vancomycin, or fidaxomicine, which are used to treat human clostridial gastrointestinal infections in Denmark. Furthermore, linezolid is not used for treatment of cases with other anaerobic bacteria in Denmark. No standard testing methods or EUCAST breakpoints for pleuromutilins or linezolid have been defined for anaerobic bacteria, and occurrence of resistance in these pathogens has yet to be explored. Due to the lack of resistance data, anaerobe bacteria such as Clostridia and *C. difficile* were not included as hazards in this risk assessment.

In enterobacteriaceae, no resistance mechanisms related to pleuromutilin have been reported, despite experimental studies suggesting a hypothetical possibility (40). Enterobacteriaceae were therefore excluded from this analysis.

Enterococci have been proven to be transmitted from either live animals or meat to man and do easily exchange resistance genes within the genera (41–43). Enterococci frequently cause urinary tract infections in humans. *Enterococcus faecalis* is also associated with serious infections such as bacteremia, surgical infections, and infectious endocarditis. *Enterococcus faecium* is usually considered a commensal with very low pathogenicity, but catheter-related infections and infectious endocarditis have been described. Serious infections with enterococci occur primarily in immunocompromised patients, and in these patients the mortality is high. Since 2013, one outbreak due to a clonal spread of a VRE *E. faecium* has been observed in hospitals in the capital region of Denmark and in the region of Zealand. The incidence of this VRE clone has been steeply increasing, which suggests that importance of linezolid in treatment will also increase in the future.

*Enterococcus* spp. were thus defined as a relevant hazard in our analysis, despite high uncertainty due to lack of surveillance data. It was included, because both resistant strains and their relevant resistance genes may spread from pigs to humans, and the consequences of development of linezolid resistance in *Enterococcus* spp. could be highly severe (30, 36). Staphylococci are known to spread from pigs to humans in persons frequently handling these animals, and extensive research has been carried out on the transmission of *S. aureus* (44–46). *S. aureus* is one of the major bacterial pathogens causing infections in humans in Denmark. The infections range from skin and soft tissue to joint and bone infections—and in more severe cases infection leads to bacteremia and infectious endocarditis. *S. aureus* accounts for 10–15% of all bacteremia in Denmark (47). In recent years, the total number of bacteremias has been slightly decreasing while bacteremias caused by staphylococci have been increasing (48, 49).

The livestock-associated MRSA CC398 is widespread in Danish swine herds and frequent transfer to humans is well documented (44–46). MRSA CC398 behaves like other *S. aureus* and was defined as a relevant hazard in the analysis, because pleuromutilin resistance has been found both on chromosomes and on plasmids. Coagulase-negative staphylococci may also be transmitted to humans but are considered a human commensal and not a significant pathogen. Resistance mechanisms may be transmitted from these to other human gram-positive bacteria. It was assumed that this mechanism is the same as for *S. aureus*. Thus, MRSA CC398 was considered a relevant hazard for the analysis, but non-MRSA staphylococci were not.

#### Conclusion for the Hazard Identification

Two pathogens were identified as relevant hazards for transferring resistance to humans due to the current and future use of pleuromutilins in Danish pigs: enterococci and MRSA CC398. Resistance may be chromosomal (staphylococci) or mobile—either carried by transposons or plasmids (staphylococci and enterococci). Anaerobe bacteria such as *Clostridium* were also described to carry resistance genes of relevance, but to date neither pleuromutilins nor linezolid are used for the treatment of infections with these bacteria. In addition, there is no surveillance of these resistance mechanisms in human anaerobe bacteria. Therefore, these hazards were not included in the risk assessment.

### Release Assessment

#### Prevalence of MRSA CC398 and Enterococci in the Danish Swine Population and Current Levels of Resistance to Pleuromutilins

In 2014, a survey was undertaken in Danish swine herds to assess the between-herd prevalence of MRSA CC398. A total of 63% of the participating breeding herds (*N* = 70) and 68% of the finishing pig herds (*N* = 205) were found to be carriers (50). No phenotypic resistance testing of the 275 MRSA CC398 isolates was undertaken.

Two Danish studies published in 2012 assessed the prevalence of MRSA CC398 in different age groups of pigs longitudinally (51, 52). The first study compared the distribution of MRSA CC398 in two herds. In one herd, MRSA CC398 was present in all sampled animals and in all age groups (sows, piglets, weaners, and finishers). In the other herd, sampled weaners and finishers were MRSA CC398 positive at all sampling occasions, while the temporal prevalence in piglets and sows was slightly lower (68–92%) (51). In the other study undertaken in six Danish swine herds, the average prevalence was higher (99%) among weaners than observed in pregnant sows (34%), piglets (77%), and finishing pigs (67%) (52).

A study conducted at the Danish National Food Institute on MRSA and methicillin-sensitive *S. aureus* (MSSA) of swine origin showed that all of MRSA CC30 strains tested and 30% of the MRSA CC398 tested were resistant to tiamulin, while the MSSA strains of CC30 and CC398 showed 46 and 38% resistance, respectively. This indicates a high prevalence of phenotypic resistance, however, the resistance mechanisms were not identified (53). In the survey performed through the Danish Veterinary and Food Adminnistration in 2014, 63% of the examined 70 breeding herds were found positive for MRSA CC398 and 68% of the 205 examined herds with finisher pigs (50). Data from the Danish swine industry’s laboratory in Kjellerup show that 61% of 36 *S. aureus* isolates from submission of diseased pigs were sensitive to pleuromutilin (Personal communication B. Svensmark, 2017). A small study on Danish mink farms showed resistance to tiamulin in 79.5% of the 39 MRSA CC398 isolates found. In Denmark, mink are fed with animal byproducts primarily consisting of slaughterhouse offal from pigs and poultry. The findings of MRSA CC398 in mink point toward transmission from pigs to mink through the feed (Personal communication K. Pedersen, DTU VET, 2017).

Resistance to pleuromutilins or linezolid is not investigated as part of DANMAP (2). According to DANMAP 2015, MRSA CC398 isolates are typically resistant to tetracycline (100%) and clindamycin (87%) with high levels of resistance to erythromycin (43%) (Table 1).

**Table 1:** **Prevalence of resistance (%)^a^ among *Enterococcus faecalis* (DANMAP 2015) and *MRSA CC398* from humans as well as swine and pork, 2014**.

Antimicrobial agent	Prevalence (%) of resistance in				
	*E. faecalis*			MRSA CC398^d^	
	Pigs^b^	Danish pork	Imported pork	Human^c^	Danish pork
Tetracycline	72	14	44	100	9
Chloramphenicol	15	4	0	n.a.	0
Ampicillin	0	0	0	n.a.	4
Erythromycin	35	6	11	43	0
Linezolid	0	0	0	0	0
TMP	n.a.	n.a.	n.a.	1	n.a.
Sulfonamide	n.a.	n.a.	n.a.	1	n.a.
Clindamycin	n.a.	n.a.	n.a.	87	n.a.
Kanamycin	n.a.	n.a.	n.a.	7	n.a.

No. of samples	40	120	27	68	23

*Source: Ref. (2)*.

*^a^Selected for antibiotic classes, which have shown to have co- and cross-resistance to pleuromutilins*.

*^b^Isolates derive from samples taken from healthy pigs at slaughter (2)*.

*^c^Isolates derived from human MRSA CC398 cases*.

*^d^Data from a point prevalence study in 2014, DANMAP 2014*.

*DANMAP, Danish Integrated Antimicrobial Resistance Monitoring and Research Program; MRSA, methicillin-resistant S. aureus; n.a., not applicable/not examined*.

Enterococci are part of the normal intestinal microbiota of pigs and exist in abundance, thus it is expected that enterococci are also found on the living pig and are ubiquitous in the stable and surroundings. The prevalence of *E. faecalis* in pig cecal content at slaughter in 2015 was 24.7% (personal communication J. Ellis-Iversen, DTU-Food, 2017).

Resistance to pleuromutilin in enterococci has not been monitored in Denmark, and no international literature on the subject was found, so the release assessment is associated with large uncertainty.

Resistance to linezolid has been monitored in *Enterococcus* spp. in Denmark since 2002, involving annual susceptibility testing of isolates from Danish pigs at slaughter and Danish and imported pork. To date, no isolates have exhibited phenotypical resistance to linezolid. In 2015, 40 isolates of *E. faecalis* from pigs were tested, and all found sensitive to linezolid (Table 2). In Table 1, resistance surveillance data in gram-positive bacteria is shown for comparison. It is noted that resistance against tetracycline (72%) and erythromycin (35%) is very common in pig isolates of *E. faecalis* (Table 1).

**Table 2:** **Results from Danish surveys of linezolid resistance in *Enterococcus faecalis* and *Enterococcus faecium* in pigs and pork, respectively**.

Year	*E. faecalis* in pigs		*E. faecalis* in pork		*E. faecium* in pigs		*E. faecium* in pork	
	Examined (no.)	Resistance (%)	Examined (no.)	Resistance (%)	Examined (no.)	Resistance (%)	Examined (no.)	Resistance (%)
2002	238	0	42	0	194	0	28	0
2003	207	0	78	0	175	0	45	0
2004	153	0	172 (12 imported)	0	148	0	52	0
2005	119	0	n.a.	n.a.	105	0	n.a.	n.a.
2006	154	0	n.a.	n.a.	145	0	n.a.	n.a.
2007	148	0	n.a.	n.a.	151	0	n.a.	n.a.
2008	149	0	197 (125 imported)	0	145	0	31 (16 imported)	0
2009	133	0	215 (109 imported)	0	151	0	39 (22 imported)	0
2010	157	0	175 (91 imported)	0	133	0	29	0
2011	117	0	178 (45 imported)	0	116	0	27	0
2012	119	0	212 (108 imported)	0	112	0	54 (22 imported)	0
2013	109	0	190 (140 imported)	0	n.a.	n.a.	53 (31 imported)	0
2014	142	0	214 (105 imported)	0	n.a.	n.a.	23	0
2015	40	0	120 (27 imported)	0	n.a.	n.a.	n.a.	n.a.

*Source: Ref. (2)*.

*n.a., not applicable/not examined*.

#### Conclusion for the Release Assessment

The overall probability of release of pleuromutilin-resistant MRSA CC398 and enterococci from pigs as a result of the use of pleuromutilins in Danish pigs was considered high for MRSA CC398 (medium uncertainty) and low for enterococci (high uncertainty).

A limited amount of data was available as pleuromutilin resistance is not systematically monitored in Denmark. The screening of a national representative pool of MRSA CC398 for mobile-resistance genes reduces the uncertainty score around the MRSA CC398 estimate to medium, but the uncertainty around the enterococci remained high due to lack of data.

The probability for cross-resistance to linezolid was considered to be medium for MRSA CC398 due to possible chromosomal point mutations (high uncertainty) and very low for enterococci (low uncertainty).

### Exposure Assessment

#### Exposure to MRSA CC398 and Enterococci *via* Meat

In Denmark, pork is the most commonly consumed type of meat. The consumption of pork per capita is 56 kg per year, followed by beef (27 kg) and poultry (26 kg). In comparison, the average consumption of pork in EU-27 is 40 kg, followed by poultry (24 kg) and beef (15 kg) (54). Most pork is eaten cooked and a minority as ready-to-eat cured products, which is expected to reduce the presence of all bacterial pathogens.

Between 2009 and 2011, Danish Veterinary and Food Administration undertook a survey for presence of MRSA CC398 in fresh meat. The average prevalence was 4% in Danish and imported pork, 1–2% in Danish and imported beef, and 15% in the imported poultry meat. The samples were analyzed qualitatively (presence/nonpresence) (55). These figures are slightly lower than the outcome of a study conducted in 2012, where 10% of the samples of Danish retail pork were found positive for MRSA CC398 (56). The Danish studies were based on qualitative enrichment methods, thus bacterial loads of MRSA CC398 in meat are not known. A study in the Netherlands found that the number of CFU/g in 75 MRSA positive meat samples were all less than 10 CFU/g (57).

Enterococci have consistently been isolated from both Danish and imported pork (2). In 2015, *E. faecium* was isolated from 6.2% and *E. faecalis* from 61.9% of 194 samples of Danish pork. Further studies on the prevalence of enterococci in food at point of consumption were on-going at the time of writing this article, but results were not yet available. The presence of enterococci in meat is considered to be independent of resistance genes and thus, humans are exposed to both resistant and sensitive enterococci through meat. Resistance to pleuromutilins is not monitored in enterococci in Denmark, but the prevalence of resistance to other antibiotics in enterococcal isolates in pork is substantially lower than in live animals (Table 1). This may suggest different subpopulations, where resistance in the latter reflects the processing environment rather than direct contamination of meat during slaughter and dressing (2). Handling of meat may lead to transmission of MRSA CC398 and *Enterococcus* spp., due to bacteria getting in contact with the skin and/or skin abrasions of the meat-handler or via cross contamination to foods, which will be eaten uncooked. Still, the probability appears to be low (58). This was confirmed in a study on the prevalence of MRSA CC398 on three Dutch slaughterhouses, which found that direct contact with live animals largely determines the carriage of MRSA CC398, although some carriers were found among slaughterhouse workers without contact with live animals (59).

The bacterial loads of *Staphylococcus* spp. and *Enterococcus* spp. are expected to decrease drastically, when pork is cooked. The only pork products consumed without heat treatment are cured and fermented products, such as dry-cured sausages. Also, here the loads of bacteria are expected to be low because of the presence of *Lactobacillus* spp. and high sodium concentrations.

Kitchen hygiene in Denmark is assessed as high due to abundant public information and effective control programs in public/commercial kitchens.

#### Exposure to MRSA CC398 and Enterococci through Direct Contact with Pigs

The main exposure pathway for MRSA CC398 is transmission through occupational contact to contaminated livestock holdings and in lesser extend subsequent transfer to their household members (60). Pigs and humans harbor different types of staphylococci primarily in their nasal cavity and secondarily on their skin. Exchange of nasal and skin flora is considered a frequent occurrence between humans and pigs and colonization of nasal mucosa with pig staphylococci is described for people having daily contact with pigs, such as farmers and veterinarians. The colonization of farmers with MRSA CC398 is well described in Denmark (60, 61).

Human carriage of MRSA CC398 in the nasal cavity may persist in a high proportion of people with daily contact to pigs. For example, Köck et al. found persistence in up to 59% of such persons (45). Persons working at livestock farms, where MRSA CC398 is present, do not only include farmers and their family members but also veterinarians, pig transporters, and slaughterhouse employees working with live animals—and the probability of transmission is considered very high (46). Contrary, single farm visits are estimated to be associated with a very low exposure risk. In 2015, surveillance data showed that 37% of human carriers did not report to have pig contact (but they could have had another animal contact), and MRSA CC398 does not appear to spread easily between humans in Denmark (61).

Resistance data in DANMAP show marked differences between resistance observed in enterococci from animals and from humans, thus no significant transmission seems to occur (2). A probable but infrequent link between pigs/pork and human enterococci has been identified (41, 42). The main transmission routes remain unknown (43).

In general, the probability of human exposure to MRSA CC398 and enterococci by direct contact with pigs in Denmark is estimated to be low, because only relatively few people have contact with live pigs. The Danish population consists of 5.7 million persons, and the Danish employed work force consisted of 2.7 million persons (2014). According to Table 3, an estimated 11,000–15,000 persons are probably carriers of MRSA CC398—corresponding to around 0.4–0.5% of the work force and 0.2–0.3% of the total population. The carriage of enterococci of animal origin farmworkers with daily contact to pigs has not been investigated.

**Table 3:** **Number of carriers of MRSA CC398 in Denmark based on information from National statistics, relevant literature and expert opinion**.

Group	No. of persons	Carrier proportion (%)	No. of carriers assuming all herds positive	No. of carriers assuming 69% positive herds^a^
Swine farmer/employee, full time	8,000^b^	74^c^	5,920	4,085
Farm employee, weekly work with swine	1,000^d^	74^e^	740	511
Swine veterinarians and advisors with daily contact to swine	200	74^e^	148	148
Craftsmen with weekly contact or less to swine	7,564^d^	11^f^	821	566
Swine transport workers	453^d^	22^g^	100	100
Abattoir workers	6,600^d^	4^h^	264	264
Household members to all persons listed above	26,199^b^	6^i^	1,511	1,073
Remaining society	5,600,000	0.10^j^	5,607	3,980
Sum of carriers		0.27	15,111	10,615

*^a^Result of a screening in 2014*.

*^b^Data from Danish Statistics, combined with best guesses among the authors validated through contact to experts*.

*^c^Unpublished data from ongoing Danish study into 10 swine herds infected with MRSA CC398. Here, 74% of the employees were positive and 10% of the household members*.

*^d^Estimates from the Danish swine industry*.

*^e^As a worst case assumption, it was assumed that weekly work with swine would result in the same carrier rate as daily work with swine, and that swine vets would have the same carrier rate as persons with daily contact to swine*.

*^f^For craftsmen with weekly contact or less, it was assumed that 74% would be positive when they leave the farm, but that they will be negative the day after—hence 74%/7 = 11%*.

*^g^Based on Van Cleef et al. (62)*.

*^h^Based on a weighted average of the prevalence observed in abattoir employees presented in Ref. (59, 62, 63)*.

*^i^Authors’ best guess based among others on unpublished data described in b. An average household has 2.1 members according to data from Danish Statistics—in the present case 1.1 persons apart from the person with contact to swine*.

*^j^Authors’ best guess*.

*MRSA, methicillin-resistant S. aureus*.

#### Other or Unknown Exposure Pathways for MRSA CC398 and Enterococci

Methicillin-resistant *S. aureus* CC398 and enterococci may spread by emission to the outside surroundings of positive swine farms. MRSA CC398 bacteria have been detected in the air and on the ground on the downwind side 150 and 300 m from positive barns, respectively. However, the probability of by-passers being contaminated with MRSA CC398 was assessed as low, while the probability of contamination through contact with soil or crops was assessed as low or unknown (55).

#### Conclusion for the Exposure Assessment

Only few Danes have direct contact to pigs. Thus, the risk of exposure to pleuromutilin-resistant MRSA CC398 (and other staphylococci of swine origin) or pleuromutilin-resistant enterococci through this route is assessed as low with low uncertainty. Other direct transmission routes are also considered to be low with medium uncertainty. For staphylococci, the probability of foodborne transmission is low (low uncertainty), whereas for enterococci, the importance of spreading through the foodborne route is unknown (high uncertainty).

### Consequence Assessment

#### Human and Economic Costs of Infection

It is essential to prevent serious infections with *S. aureus* and enterococci and to secure effective treatment options through the control of multidrug-resistant bacteria. Although the human pathogenicity of MRSA CC398 and resistant *Enterococcus* spp. is low, it is considered important to prevent the spread of these bacteria, especially to weakened and hospitalized patients. The costs associated with screening, isolation, and treatment of patients with clinical infections due to MRSA CC398 was estimated in Denmark in 2015. For patients admitted to intensive care, the expense was estimated to increase twofold due to extra precautions, prolonged hospital stay, and more complications, amounting to approximately €27,000 per patient (64).

#### Increased Public Health Burden and Disease due to Consumption of Pleuromutilin in Pigs

A retrospective Danish study of human MRSA CC398 isolates from 1999 to 2011 showed that infections were primarily associated with skin and soft tissues and other noninvasive infections (60). The same study reported the incidence of MRSA CC398 infections in 2011 to be 1.1 per 100.000 person-years. In Denmark, human MRSA is notifiable, and clinical MRSA isolates are referred mandatorily to the reference laboratory at Statens Serum Institut. In 2012, the Danish Health and Medicines Authority published a revised MRSA guideline, which introduced contact with pigs as a new risk factor for carriage of the bacteria. Therefore, persons working with pigs and household members of such persons are tested for MRSA routinely on admission to hospital. This has given a greater share of findings of healthy carriers and has contributed to effective control of spread at Danish hospitals (60). In 2015, a total of 1,173 MRSA CC398 carriers were detected through the screening program (39% of all described human MRSA carriers) (48). In all, 208 were clinical infections, and of these 3 were bacteremia cases—corresponding to 3/1.173 = 0.3% of the identified carriers. Throughout the period from 2007 to 2016, the share of cases with clinical infection in Denmark was lower for MRSA CC398 than that observed for the remaining types of MRSA (Table 4).

**Table 4:** **Human cases of bacteremia and death in Denmark, distributed according to type of staphylococci, for the time period 2011 to mid-2016**.

Kind of staphylococci	Year						30-day mortality (%)
	2011	2012	2013	2014	2015	2016^a^	
MSSA bacteremia	1,504	1,507	1,735	1,908	1,973	^b^	
MSSA deaths	347	337	408	425	452	^b^	23
Non-CC398 MRSA bacteremia	20	19	26	48	26	13	
Non-CC398 MRSA deaths	6	4	6	10	6	3	23
CC398 MRSA bacteremia	1	2	4	8	3	5	
CC398 MRSA deaths	0	1	2	2	1	0	26

*Source: annual report from Statens Serum Institut (www.ssi.dk)*.

*^a^Only data from the first 6 months of 2016 were available*.

*^b^Figures for MSSA 2016 were not available by February 2017*.

*MSSA, methicillin-sensitive S. aureus; MRSA, methicillin-resistant S. aureus*.

Resistance testing of all human MRSA is performed at the Clinical Microbiological Departments for treatment purposes only, and data from these are thus not officially reported. At Statens Serum Institut, a share of the referred clinical strains is tested with the TREK microtiter system. None were found resistant to linezolid (Table 1) (2). Thus, none of the examined human cases with MRSA CC398 could be attributed directly to pleuromutilin resistance. In theory, selected mutants of *S. aureus* resistant to linezolid can exhibit cross-resistance to tiamulin (17). However, data on linezolid resistance are a poor indicator of the presence or absence of pleuromutilin resistance due to only partly overlaps of resistance mechanisms.

*Enterococcus faecalis* is the frequent cause of urinary tract infections. These are usually mild but may be recurrent especially in the elderly and in patients with urinary catheters. In some of these patients, bacteremias and endocarditis develop. Resistance testing on enterococcal urinary strains does not include testing for linezolid, thus the burden due to consumption of pleuromutilins could not be estimated but it was judged to be negligible.

#### Prolonged Human Illness and/or Death due to Consumption of Pleuromutilin in Pigs

Treatment failure was defined as prolonged human illness and/or death due to resistance to pleuromutilins.

In 2015, the incidence of bacteremia with presence of *S. aureus* in Denmark was estimated to be 36.7 cases per 100,000 inhabitants, corresponding to 1,973 bacteremia cases (2). MRSA accounted for 29 of these, and 3 of these were MRSA CC398 (Table 4). Until mid-2016 altogether six deaths with MRSA CC398 have been reported (based among others on reference 48 including corrections presented in Table 4). All of these suffered from severe comorbidity or prolonged illness. In general, the 30-day mortality due to staphylococcal bacteremia is high, for 2011 to 2016, the 30-day mortality was 23% for MSSA and 26% for MRSA (Table 4) (2).

A Danish study published in 2013 estimated the disease burden due to severe enterococcal infections (65). The incidence was found to be increasing during recent years and was estimated to 19.6/100,000 person-years. Most enterococcal bacteremias were found to be associated with hospitalization. Although *E. faecalis* was mainly associated with urinary tract infections as the primary cause of bacteremia, *E. faecium* was primarily associated with abdominal surgery. The 30-day mortality was high for patients with bacteremias—21.4% in patients with *E. faecalis* and 34.6% in patients with *E. faecium*.

Only a fraction of enterococcal isolates from blood cultures will usually be tested for sensitivity to linezolid. At Rigshospitalet (a highly specialized tertiary hospital in the Capital Region), all clinical enterococcal strains (*N*=1,715) isolated from November 1, 2015, to November 1, 2016, were tested for phenotypical resistance to linezolid. Of the 41 linezolid resistant strains, six were sent to the reference laboratory at Statens Serum Institut for whole-genome sequencing. In one linezolid-resistant *E. faecalis*, an *optrA* gene was found. This belonged to the sequence type (ST) 16, a ST that has previously been associated with pigs (Personal communication, A. Hammerum, Statens Serum Institut, 2017).

Although surveillance on the spread of clinical VRE exist, based on voluntary referral from the Danish Clinical Microbiological Departments to the reference laboratory at Statens Serum Institut, this does not include registration of prolonged illness or deaths due to these.

None of the approximately 300 clinical VRE submitted to Statens Serum Institut for whole-genome sequencing carried the *optrA* gene (Personal communication, A. Hammerum, Statens Serum Institut, 2017). The strains were not examined for the presence of other transmissible genes but the resistance profiles of the tested strains suggested that none cfr-carrying strains were among them.

It is difficult to establish whether the high mortality of staphylococcal and enterococcal bacteremias would be influenced by an additional resistance to pleuromutilins or linezolid. For vulnerable groups—such as hospitalized and immunocompromised—the probability of negative consequences will be high, if the general resistance pressure increases. The risk for acquiring an infection with a multiresistant strain increases with increased length of stay and in patients who are admitted to intensive care or have undergone abdominal surgery. This often leads to long-lasting catheterization and the risk of colonization of indwelling catheters with multiresistant bacteria. These patients will often be treated with several antimicrobial courses, and this eventually causes a shift in the microbiological flora from more sensitive toward more resistant strains. Prolonged antibiotic treatment will also select for already resistant bacteria among the microbiome of the patient.

For patients suffering from infections with *E. faecalis* or *E. faecium*, treatment failure mainly occurs in severely ill patients with bacteremia, significant comorbidities, and long hospitalization. For staphylococcal infections, treatment failure is mainly seen in complicated cases of bacteremia with no established primary focus for the infection or in bone infections, where the infecting strain might adjust to the human host and show slow or no response to the antimicrobial treatment.

For these very vulnerable patient groups with multidrug resistant bacteria, linezolid and the anticipated new pleuromutilin for systemic treatment may constitute a relevant antimicrobial to consider. In addition, the consequences due to already existing pleuromutilin- or linezolid-resistant strains will be severe for the individual patient.

Today, the prevalence of multidrug-resistant gram-positive human infections in Denmark in general is very low (2) and very few patients are treated with linezolid. No treatment failure due to linezolid has been reported in Denmark so far.

#### Partial Conclusion for the Consequence Assessment

Human carriage of MRSA CC398 and urinary tract infections with enterococcal strains are fairly common, whereas severe infections with MRSA CC398 happen only rarely and severe infections with enterococci are mainly associated with prolonged hospitalization. Pleuromutilin is not used for humans in Denmark, and linezolid is only used rarely, in specific hospital settings and in selected patient groups. However, linezolid is a last line drug reserved for the few infections, where other antimicrobials cannot be used. Therefore, the consequence assessment for MRSA CC398 was assessed as very low probability for the public in general, but high for vulnerable groups (low uncertainty), whereas for pleuromutilin resistant *Enterococcus*, it was assessed that there was a low probability of adverse consequences across the population (high uncertainty).

## Discussion

### Risk Estimate

**Figure 2:**
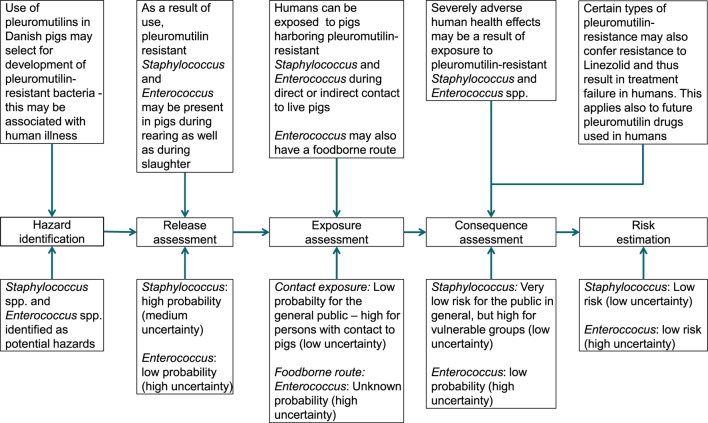
**Summary of risk assessment for human health related to use of pleuromutilins in Danish pigs**.

The current risk for human health-associated consumption of pleuromutilins in Danish swine was assessed as low under the current conditions. This is an increase compared to the previous assessment conducted in 2010, where a very low risk was found (7). A summary of the different elements of the risk assessment is presented in Figure 2.

The consumption of pleuromutilins in Denmark is low compared to other European countries (9) and has not changed markedly over the past 20 years. It may be considered a controversy to call a use involving 8,000 kg of antimicrobials in livestock low, yet it should be taken into account that the annual production of pigs in Denmark is 28 million (compared to a human population size of close to 6 million). The relative low use is due to awareness of and focus on the antimicrobial resistance issues in Denmark and strict prescription and usage policies prohibiting both use as growth promoter and preventive use of antibiotics in animals.

Among these policies, the Yellow Card Scheme has shown to have substantial influence on the overall consumption of antimicrobials in pigs. The newest version of the scheme differentiates between weighing of different antibiotic groups: a farmer using, e.g., fluoroquinolones will reach the allowed limit for the herd very quickly, since the consumption of fluoroquinolones is multiplied by 10 when the average consumption per pig is calculated. The weights for tetracyclines are currently 1.2 (and by 1.5 from September 30, 2017) and 0.95 for simple penicillin, sulfonamides, trimethoprim, and pleuromutilins (5). The allowed limits are repeatedly adjusted, and from March 2017 the limit for consumption for sows with piglets were reduced to 4.1 weighted ADD (ADD_w_) per 100 animals per day, for weaners to 21.8 ADD_w_ per 100 animals per day, and for finishing pigs to 5.6 ADD_w_ per 100 animals per day (4).

In general, the need for antimicrobial treatment of Danish pigs may be lower than in many other countries, because dysentery has been eradicated from the majority of swine herds. Pig health is high in the Danish pig production due to a widely used SPF Certification scheme. The SPF sow herds produce piglets, boars, and gilts for almost all production herds, and the majority of the sow herds are SPF certified with public access to data describing the sow herds’ infection status. Hereby, finishing pig producers can purchase replacement animals with the same infection status as in their own herd, minimizing the risk of introduction of additional unwanted pathogens.

The hazard identification showed that pleuromutilin resistance may be transmitted from pigs to humans in various ways and by different kinds of bacteria, but it also showed that the knowledge about the importance of these ways was limited making it difficult to distinguish potential hazards from risks.

The release assessment showed that pleuromutilin resistance is present in MRSA CC398 in non-negligible levels in Denmark, suggesting a potential risk pathway to humans. This may have a negative impact on especially immunocompromised persons, if cross-resistance to linezolid becomes more prevalent.

The exposure assessment showed that despite that MRSA CC398 is present in the majority of the Danish swine herds, only a small proportion of the human population are carriers of MRSA CC398. This number might be increasing since one-third of the found carriers were not associated with regular contact to swine and thus might constitute a pool of exposure to vulnerable persons not covered by the existing screening guidelines, and hence the probability of negative consequences may increase. The presence of enterococci in meat was high, but appropriate handling and cooking reduce the foodborne exposure to low. Very little is known about the human exposure of *Enterococcus* spp. through direct contact with pigs, but since a low proportion of Danes are exposed to living pigs, it was also assessed as low.

It is difficult to estimate the additional burden on severe disease and death due to pleuromutilin- or linezolid-resistant MRSA CC398 and enterococci, since death by these bacteria often occurs in already severely ill patients and at a late stage in hospitalization. It is difficult to judge whether possible additional resistance mechanisms in these patients contribute to the increased mortality observed, selected due to prolonged antimicrobial treatment.

Several attempts have been made to estimate the number of significant human disease and deaths due to resistant bacteria. In 2009, the European Center for Disease Control (ECDC) published a report on the disease burden due to resistant bacteria based on European surveillance data from 2007 (66). The report estimated the number of additional deaths due to resistant bacteria to be 25,000 annually. The estimate was based on a calculated total number of 171,200 bacteremias with MRSA causing an estimated 5,400 deaths and 18,100 bacteremias with vancomycin-resistant *E. faecium* causing 1,500 deaths. The McCabe score for modeling the expected extra deaths occurring from these infections (67) could not be used since information on pleuromutilin resistance is lacking in the clinical strains from bacteremia and since the relevant bacteria are already multiresistant. We assumed that the presence of the pleuromutilin-resistance mechanism itself does not contribute to more severe disease or death. Especially since pleuromutilins are not used in human treatment.

Still, it must be assumed that the use of pleuromutilins will increase the release of MRSA CC398 and resistant enterococci from pigs to a certain degree, as will the consumption of other antimicrobials conferring coresistance to pleuromutilins.

In Denmark, multiresistance in human pathogens is not endemic, and screening procedures and infection control measures at hospitals are very effective. This, combined with the fact that MRSA CC398 does not seem to have a high speed of transmission from pigs to humans or between humans, makes it plausible to estimate the risk of impact on human health to be altogether low. This also applies to multiresistant enterococci, where no significant transmission from pigs to humans yet has been described. However, a theoretical possibility of transmission of resistance genes does exist.

### Uncertainty and Its Implications for Predictions into the Future

The assessment was associated with some uncertainty due to lack of data and is very dependent on the current very low occurrence of mobile, genetic resistance mechanisms, low incidence of multidrug-resistant bacteria in the Danish population in general, and a very rare need for “last choice drugs” in severely ill patients. While the Danish surveillance system for many years has covered bacteria with known human pathogenicity, surveillance on other than known human pathogen bacteria such as commensals, and the occurring phenotypic resistance and resistance mechanisms in these is missing.

The analysis showed that resistance transfer between species is complex and multifactorial. It is unlikely that all transmission pathways are known to us today. Sporadic findings are often reported, but lack of repeatability and data restricts the ability to assess how important such findings are and whether they pose a risk to public health. This also includes the lack of surveillance of resistance in anaerobe bacteria. The gram-positive anaerobe gut flora in pigs and humans might constitute a pool for resistance mechanisms, which constantly exchanges with other bacteria, including the ones that more frequently cause human disease.

The increased use of whole-genome sequencing is likely to provide additional valuable knowledge on transfer and potential resistance risks, but the link to phenotypic and clinical resistance and, thus, importance for public health remain to be elucidated.

Mathematical modeling is a valuable tool in simulating pathways and identifying ways of reducing resistance. However, the relationship between reducing consumption of antibiotics and reducing resistance is not necessarily linear and still needs to be established for modeling to have full value. This includes among others the fact that some resistance mechanisms are unlikely to reverse if they are associated with increased fitness for the bacteria (68, 69). Furthermore, data and knowledge gaps cause problems in modeling too and enhance uncertainty around outputs.

When new relevant data become available indicating that there might be a change in resistance prevalence or consumption patterns, the present risk assessment should be repeated. This is especially important if the consumption of pleuromutilins increases substantially, and this leads to a higher prevalence of mobile, easily transferrable resistance mechanisms of importance to human health, or if linezolid and pleuromutilins becomes more important in human medicine.

### The Choice of Risk Assessment Framework

The risk related to antimicrobial resistance may be assessed in different ways. One simple way may be to use the WHO ranking of critically important antimicrobials, which in essence is based on the worldwide need for treatment in human medicine (70). The drawback of this approach is that it does not take into account differences between countries in antimicrobial consumption and resistance, meaning that critically important antimicrobials will not necessarily be critically important everywhere. In addition, it does not take into account whether surveillance or any data from research show that a specific veterinary use is associated with a non-negligible risk for humans in a given country or region.

We were inspired by the EMA framework, which includes the risk pathway in details; from the use of pleuromutilin in pigs to the consequences in humans. Initially, we followed the EMA framework very stringent, in the exact order and in details for the many elements listed. This systematic approach was very effective to point out important gaps in the surveillance and knowledge of transmission routes and rates (8). However, the framework was too comprehensive for our purpose, led to a plethora of details on the expense of the overview, and thus was abandoned. While the EMA framework was developed for introduction of a new drug on the market with unknown mechanisms and risks, our study was assessing an established antibiotic with existing—albeit not perfect—surveillance data. The complexity of the EMA framework added unnecessary areas of uncertainty and complicated communication of the results to relevant audience of our assessment.

In addition, the EMA framework does not specify how the hazard identification should be undertaken and which criteria should be used to distinguish hazards from potential risks, for which a risk assessment is required. Should the selection, e.g., be based solely on resistance mechanisms where treatment failure is observed or is presence of resistance in bacteria from animals in itself a sufficient argument? Moreover, should the hazard identification be based on national or international findings? We decided to use international data for the hazard identification and national data for the release, exposure, and consequence assessment. We also decided to focus on the different ways in which resistance can be dispersed, and here the bacteria may be seen as a vehicle, not the hazard in itself. Moreover, we found that the EMA framework was most appropriate for resistance forms that are bound to specific bacteria with well-described transmission routes from animal to humans. In the case of transmission of mobile resistance genes, which may travel between different bacterial species at high speed, the EMA framework suffers from being insufficient, especially because the bacteria species is considered the hazard, not the drug use or the resistance genes.

The effect on the environment was not assessed as this is outside the scope of the framework developed by EMA. In line, off-label use—including misuse—was not included in the analysis, but it is considered a very minor issue in Denmark. These shortcomings and limitations could be addressed in future research.

As far as we are aware, this is the first public-accessible publication based on the framework developed by EMA for the assessment of the public health risk related to use of antimicrobials in livestock. The output is a risk assessment, which is one part of the risk analysis process. Other elements are risk management and risk communication. EMA states that this guidance aims to provide a systematic approach to the evaluation of the associated scientific data and to improve the transparency and consistency of the regulatory decision-making process—we agree that the guideline can be used for this purpose but that flexibility should be applied to ensure logic and easy communication to the relevant audience.

### Recommendation

Care should be taken to ensure a prudent use of antibiotics in livestock and only for treatment of disease, metaphylaxis, and planned eradication. Moreover, long-term treatments should be avoided. This will ensure that pleuromutilins will remain effective for the treatment of swine also in the future. In line, swine dysentery should be eradicated, because this will reduce the need for treatment (9).

For most indications for which pleuromutilins are authorized, tetracyclines and macrolides can replace pleuromutilins, except for dysentery. But these antimicrobial classes are not perceived as having a lower risk for human health than pleuromutilins. Therefore, pleuromutilins were still recommended by the working group for sensible use in Danish swine for treatment of the relevant infection.

The newest changes to the Yellow Card scheme are likely to lead to a slightly higher use of pleuromutilins but to a lower use of other antimicrobial classes that coselect for pleuromutilin resistance (4). It is difficult to predict the consequences of this management decision, which was implemented just before this analysis was done. In January to November 2016, the increase in consumption of pleuromutilin was limited to 0.3% compared to the same months in 2015. However, the consumption may increase in the future, and it emphasizes the need to monitor on the consumption of pleuromutilins and their resistance mechanisms in Danish pigs and to reassess the risk, if any risk factors to human health changes.

In line with EMA, we recommend the establishment of routine monitoring of pleuromutilin resistance in relevant swine bacteria to develop an early warning system (9). Pleuromutilin resistance is not monitored systematically in Denmark, and therefore, it is extremely difficult to estimate the contribution to the prevalence of MRSA CC398 and enterococci within pig farms and the prevalence of resistance mechanisms.

As resistance in both human and swine pathogens seems to be emerging, there is a need for targeted surveillance of specific resistances. Focus on *Staphylococci* spp.—including a genetic component to search for resistance genes—is recommended. At present, there is no systematic monitoring of *Staphylococcus* spp. or MRSA in pigs in Denmark. *Enterococcus* spp. are monitored as part of the program for monitoring of resistance to erythromycin, linezolid, and chloramphenicol—but this does not involve monitoring for resistance to pleuromutilins. The emergence of VRE in hospital settings is of special concern. A program for surveillance is being established in collaboration between clinical microbiological departments and the Danish health authorities. This surveillance program is expected to cover the emerging resistance for linezolid as well. In the existing collaboration between the veterinary authorities, DTU FOOD, and Statens Serum Institut, the establishment of a more thorough surveillance of resistance mechanisms is being discussed.

## Conclusion

The current risk for human health associated with consumption of pleuromutilins in Danish swine was assessed as low under the current conditions. This is an increase compared to the previous assessment where a very low risk was found.

Two hazards were identified: MRSA CC398 and enterococci. For MRSA CC398, the total risk was estimated as low (low uncertainty). For enterococci, the total risk was estimated as low (high uncertainty).

The assessment should be repeated, if pleuromutilin consumption increases substantially, resulting in increased prevalence of mobile, easily transmissible resistance mechanisms, or if the need for linezolid as a last line human drug increases. Continuous monitoring of pleuromutilin resistance in selected human pathogens should therefore be considered.

## Author Contributions

LA, MA, and US contributed to the design of the work, the risk assessment, and the drafting of the manuscript. JE-I contributed to the framework development and writing of the manuscript. JD contributed to the risk assessment.

## Conflict of Interest Statement

The authors JE-I and US certify that they have no affiliations with or involvement in any organization or entity with any financial interest or non-financial interest in the subject matter or materials discussed in this manuscript. The authors LA and JD are affiliated with an institution, which gives advices to Danish farmers and the agro-food-business. MA is affiliated with an organization, which gives advice to the veterinary pharmaceutical industry in Denmark.

## References

[1] Stege H Bager F Jacobsen E Thougaard A VETSTAT – the Danish system for surveillance of the veterinary use of drugs for production animals. Prev Vet Med (2003) 57:105–15. 10.1016/S0167-5877(02)00233-7 12581594

[2] DANMAP. Use of Antimicrobial Agents and Occurrence of Antimicrobial Resistance in Bacteria from Food Animals, Foods and Humans in Denmark. Copenhagen: Danish Veterinary Institute, Danish Veterinary and Food Administration, Statens Serum Institute and Danish Medicines Agency (2015).

[3] Alban L Dahl J Andreasen M Petersen J Sandberg M Possible impact of the “yellow card” antimicrobial scheme on meat inspection lesions in Danish finisher pigs. Prev Vet Med (2013) 108:334–41. 10.1016/j.prevetmed.2012.11.010 23194892

[4] Danish Veterinary and Food Administration. Permit Limits for Consumption of Antimicrobials (in Danish). (2016). Available from: https://www.foedevarestyrelsen.dk/Leksikon/Sider/Grænseværdier-for-antibiotikaforbrug-og-dødelighed.aspx

[5] Danish Veterinary and Food Administration. Weighted Animal Daily Dose (in Danish). (2016). Available from: https://www.foedevarestyrelsen.dk/Leksikon/Sider/Vægtede-ADDer.aspx

[6] Alban L Nielsen EO Dahl J. A human health risk assessment for macrolide-resistant *Campylobacter* associated with the use of macrolides in Danish pig production. Prev Vet Med (2008) 83:115–29. 10.1016/j.prevetmed.2007.06.006 17659797

[7] DANMAP. Use of Antimicrobial Agents and Occurrence of Antimicrobial Resistance in Bacteria from Food Animals, Food and Humans in Denmark. Copenhagen: Danish Veterinary Institute, Danish Veterinary and Food Administration, Statens Serum Institute and Danish Medicines Agency (2010). 158 p.

[8] European Medicines Agency. Guideline on the Assessment of the Risk to Public Health from Antimicrobial Resistance due to the Use of an Antimicrobial Veterinary Medicinal Product in Food-Producing Animals. London, United Kingdom: European Medicines Agency (2015). EMA/CVMP/AWP/706442/2013.

[9] European Medicines Agency. Reflection Paper on Use of Pleuromutilins in Food-Producing Animals in the European Union: Development of Resistance and Impact on Human and Animal Health. London, United Kingdom: European Medicines Agency (2013). EMA/CVMP/AWP/119489/2012.

[10] ECDC/EFSA/EMA. ECDC/EFSA/EMA first joint report on the integrated analysis of the consumption of antimicrobial agents and occurrence of antimicrobial resistance in bacteria from humans and food-producing animals. EFSA J (2015) 13(1):114 10.2903/j.efsa.2015.4006

[11] Hogenauer G Tiamulin and pleuromutilin. In: Hahn F editor. Chapter Mechanism of Action of Antibacterial Agents. Volume 5/1 of the Series Antibiotics. Berlin, Germany: Springer-Verlag (1979). p. 344–60.

[12] Shang R Wang J Guo W Liang J. Efficient antibacterial agents: a review of the synthesis, biological evaluation and mechanism of pleuromutilin derivatives. Curr Top Med Chem (2013) 13(24):3013–25. 10.2174/15680266113136660217 24200363

[13] Danish Medicines Agency. Product Resume for Tiamulin. (2016). Available from: http://www.produktresume.dk/docushare/dsweb/View/Collection-72

[14] European Medicines Agency. Product Profile for Valnemulin. (2004). Available from: http://www.ema.europa.eu/docs/en_GB/document_library/EPAR_-_Scientific_Discussion/veterinary/000042/WC500064379.pdf

[15] DANMAP. Use of Antimicrobial Agents and Occurrence of antimicrobial Resistance in Bacteria from Food animals, Food and Humans in Denmark. Copenhagen: Danish Veterinary Institute, Danish Veterinary and Food Administration, Statens Serum Institute and Danish Medicines Agency (2011). 136 p.

[16] NASDAQ. Nabriva Initiates Second Phase 3 Clinical Trial of Lefamulin (“LEAP 2”) in Patients with Community-Acquired Bacterial Pneumonia (CABP). (2016). Available from: https://globenewswire.com/news-release/2016/04/11/827513/0/en/Nabriva-Initiates-Second-Phase-3-Clinical-Trial-of-Lefamulin-LEAP-2-in-Patients-with-Community-Acquired-Bacterial-Pneumonia-CABP.html.

[17] Miller K Dunsmore CJ Fishwick CWG Chopra I. Linezolid and tiamulin cross-resistance in *Staphylococcus aureus* mediated by point mutations in the peptidyl transferase center. Antimicrob Agents Chemother (2008) 52:1737–42. 10.1128/AAC.01015-07 18180348 PMC2346621

[18] Gentry DR Rittenhouse SF McCloskey L Holmes DJ. Stepwise exposure of *Staphylococcus aureus* to pleuromutilins is associated with stepwise acquisition of mutations in rplC and minimally affects susceptibility to retapamulin. Antimicrob Agents Chemother (2007) 51(6):2048–52. 10.1128/AAC.01066-06 17404009 PMC1891380

[19] Karlsson M Gunnarsson A Franklin A Susceptibility to pleuromutilins in *Brachyspira (Serpulina) hyodysenteriae*. Anim Health Res Rev (2007) 2(1):59–66. 10.1079/AHRR200118 11708748

[20] Lobová D Smola J Cizek AJ. Decreased susceptibility to tiamulin and valnemulin among Czech isolates of *Brachyspira hyodysenteriae*. J Med Microbiol (2004) 53(Pt 4):287–91. 10.1099/jmm.0.05407-0 15017284

[21] Pringle M Landén A Unnerstad HE Molander B Bengtsson B. Antimicrobial susceptibility of porcine *Brachyspira hyodysenteriae* and *Brachyspira pilosicoli* isolated in Sweden between 1990 and 2010. Acta Vet Scand (2012) 54:54. 10.1186/1751-0147-54-54 22998753 PMC3526423

[22] Schwendener S Perreten V. New transposon Tn6133 in methicillin-resistant *Staphylococcus aureus* ST398 contains vga (E), a novel streptogramin A, pleuromutilin, and lincosamide resistance gene. Antimicrob Agents Chemother (2011) 55:4900–4. 10.1128/AAC.00528-11 21768510 PMC3187003

[23] Wendlandt S Kadlec K Fessler AT Schwarz S. Identification of ABC transporter genes conferring combined pleuromutilin-lincosamide-streptogramin A resistance in bovine methicillin-resistant *Staphylococcus aureus* and coagulase-negative staphylococci. Vet Microbiol (2015) 177:353. 10.1016/j.vetmic.2015.03.027 25891423

[24] Long KS Poehlsgaard J Kehrenberg C Schwarz S Vester B. The Cfr rRNA methyltransferase confers resistance to phenicols, lincosamides, oxazolidinones, pleuromutilins, and streptogramin A antibiotics. Antimicrob Agents Chemother (2006) 50:2500–5. 10.1128/AAC.00131-06 16801432 PMC1489768

[25] Deshpande LM Ashcraft DS Kahn HP Pankey G Jones RN Farrell DJ Detection of a new cfr-like gene, cfr(B), in *Enterococcus faecium* isolates recovered from human specimens in the United States as part of the Sentry antimicrobial surveillance program. Antimicrob Agents Chemother (2015) 59(10):6256–61. 10.1128/AAC.01473-15 26248384 PMC4576063

[26] Kehrenberg C Aarestrup FM Schwartz S. IS*21-558* insertion sequences are involved in the mobility of the multiresistance gene cfr. Antimicrob Agents Chemother (2007) 51:779–81. 10.1128/AAC.01340-06 17145796 PMC1797725

[27] Kehrenberg C Cuny C Strommenger B Schwarz S Witte W. Methicillin-resistant and -susceptible *Staphylococcus aureus* strains of clonal lineages ST398 and ST9 from swine carry the multidrug resistance gene cfr. Antimicrob Agents Chemother (2009) 53(2):779–81. 10.1128/AAC.01376-08 19047652 PMC2630595

[28] Hansen LH Vester B. A cfr-like gene from *Clostridium difficile* confers multiple antibiotic resistance by the same mechanism as the cfr gene. Antimicrob Agents Chemother (2015) 59(9):5841–3. 10.1128/AAC.01274-15 26149991 PMC4538495

[29] Liu Y Wang Y Wu C Shen Z Schwarz S Du XD First report of the multidrug resistance gene cfr in *Enterococcus faecalis* of animal origin. Antimicrob Agents Chemother (2012) 56(3):1650–4. 10.1128/AAC.06091-11 22203597 PMC3294887

[30] Wendlandt S Lozano C Kadlec K Gomez-Sanz E Zarazaga M Torres C The enterococcal ABC transporter gene *lsa*(E) confers combined resistance to lincosamides, pleuromutilins and streptogramin A antibiotics in methicillin-susceptible and methicillin-resistant *Staphylococcus aureus*. J Antimicrob Chemother (2013) 68:473 10.1093/jac/dks398 23047809

[31] Wendlandt S Shen J Kadlec K Wang Y Li B Zhang WJ Multidrug resistance genes in staphylococci from animals that confer resistance to critically and highly important antimicrobial agents in human medicine. Trends Microbiol (2015) 23(1):44–54. 10.1016/j.tim.2014.10.002 25455417

[32] Kadlec K Schwarz S. Novel ABC transporter gene, vga(C), located on a multiresistance plasmid from a porcine methicillin-resistant *Staphylococcus aureus* ST398 strain. Antimicrob Agents Chemother (2009) 53(8):3589–91. 10.1128/AAC.00570-09 19470508 PMC2715595

[33] Kadlec K Fessler AT Hauschild T Schwarz S. Novel and uncommon antimicrobial resistance genes in livestock-associated methicillin-resistant *Staphylococcus aureus*. Clin Microbiol Infect (2012) 18(8):745–55. 10.1111/j.1469-0691.2012.03842.x 22509728

[34] WHO. WHO Alert: Identification of Enterococcal Isolates of Food and Human Origin Carrying the Linezolid-Resistance OptrA Gene. Copenhagen: IHR National Focal Point of Denmark (2016).

[35] Stefani S Bongiorno D Mongelli RE Campanile F. Linezolid resistance in staphylococci. Pharmaceuticals (Basel) (2010) 3:1988–2006. 10.3390/ph3071988 27713338 PMC4036669

[36] Wang Y Lv Y Cai J Schwarz S Cui L Hu Z A novel gene, optrA, that confers transferable resistance to oxazolidinones and phenicols and its presence in *Enterococcus faecalis* and *Enterococcus faecium* of human and animal origin. J Antimicrob Chemother (2015) 70(8):2182–90. 10.1093/jac/dkv116 25977397

[37] Li D Wang Y Schwarz S Cai J Fan R Li J Co-location of the oxazolidinone resistance genes optrA and cfr on a multiresistance plasmid from *Staphylococcus sciuri*. J Antimicrob Chemother (2016) 71(6):1474–8. 10.1093/jac/dkw040 26953332

[38] WHO. WHO Alert: Foodborne Enterococcus faecalis Carrying the Linezolid-Resistant OptrA Gene. Bogota: IHR National Focal Point of Colombia (2016).

[39] Swedres-Svarm. Consumption of Antibiotics and Occurrence of Antibiotic Resistance in Sweden. Solna/Uppsala: Public Health Agency of Sweden and National Veterinary Institute (2015). 123 p.

[40] Long KS Hansen LH Jakobsen L Vester B. Interaction of pleuromutilin derivatives with the ribosomal peptidyl transferase center. Antimicrob Agents Chemother (2006) 50(4):1458–62. 10.1128/AAC.50.4.1458-1462.2006 16569865 PMC1426994

[41] Larsen J Schønheyder HC Lester CH Olsen SS Porsbo LJ Garcia-Migura L Porcine-origin gentamicin-resistant *Enterococcus faecalis* in humans, Denmark. Emerg Infect Dis (2010) 16(4):682–4. 10.3201/eid1604.090500 20350387 PMC3321936

[42] Larsen J Schønheyder HC Singh KV Lester CH Olsen SS Porsbo LJ Porcine and human community reservoirs of *Enterococcus faecalis*, Denmark. Letter to the editor. Emerg Infect Dis (2011) 17(12): 2395–7. 10.3201/eid1712.101584 22172303 PMC3311169

[43] Donabedian SM Thal LA Hershberger E Perri MB Chow JW Bartlett P Molecular characterization of gentamicin-resistant enterococci in the United States: evidence of spread from animals to humans through food. J Clin Microbiol (2003) 41:1109–13. 10.1128/JCM.41.3.1109-1113.2003 12624037 PMC150269

[44] Garcia-Graells C van Cleef BA Larsen J Denis O Skov R Voss A. Dynamic of livestock-associated methicillin-resistant *Staphylococcus aureus* CC398 in pig farm households: a pilot study. PLoS One (2013) 8(5):e65512. 10.1371/journal.pone.0065512 23741497 PMC3669288

[45] Köck R Schaumburg F Mellmann A Köksal M Jurke A Becker K Livestock-associated methicillin-resistant *Staphylococcus aureus* (MRSA) as causes of human infection and colonization in Germany. PLoS One (2013) 8(2):e55040 10.1371/journal.pone.0055040 23418434 PMC3572123

[46] Crombé F Argudín MA Vanderhaeghen W Hermans K Haesebrouck F Butaye P. Transmission dynamics of methicillin-resistant *Staphylococcus aureus* in pigs. Front Microbiol (2013) 4:57. 10.3389/fmicb.2013.00057 23518663 PMC3602589

[47] Benfield T Espersen F Frimodt-Møller N Jensen AG Larsen AR Pallesen LV Increasing incidence but decreasing in-hospital mortality of adult *Staphylococcus aureus* bacteraemia between 1981 and 2000. Clin Microbiol Infect (2007) 13(3):257–63. 10.1111/j.1469-0691.2006.01589.x 17391379

[48] Statens Serum Institute. MRSA 2015. EPI-NEWS Week 23. (2016). Available from: http://www.ssi.dk/Aktuelt/Nyhedsbreve/EPI-NYT/2016/Uge%2023%20-%202016.aspx

[49] Nielsen SL Pedersen C Jensen TG Gradel KO Kolmos HJ Lassen AT. Decreasing incidence rates of bacteremia: a 9-year population-based study. J Infect (2014) 69(1):51–9. 10.1016/j.jinf.2014.01.014 24576825

[50] Anonymous. MRSA Risk Assessment Prepared by the MRSA Expert Group. The Danish Veterinary and Food Administration (2014). 46 p. Available from: https://www.foedevarestyrelsen.dk/english/SiteCollectionDocuments/Dyresundhed/Rapport_fra_MRSA-ekspertgruppe%20EN.pdf

[51] Broens EM Espinosa-Gongora C Graat EAM Vendrig N Van Der Wolf PJ Guardabassi L Longitudinal study on transmission of MRSA CC398 within pig herds. BMC Vet Res (2012) 8:58. 10.1186/1746-6148-8-58 22607475 PMC3532224

[52] Espinosa-Gongora E Larsen J Moodley A Nielsen JP Skov RL Andreasen M Farm-specific lineages of methicillin-resistant *Staphylococcus aureus* clonal complex 398 in Danish pig farms. Epidemiol Infect (2012) 140(10):1794–9. 10.1017/S0950268811002391 22117120

[53] Seier-Petersen MA Nielsen LN Ingmer H Aarestrup FM Agersø Y. Biocide susceptibility of *Staphylococcus aureus* CC398 and CC30 isolates from pigs and identification of the biocide resistance genes, *qacG* and *qacC*. Microb Drug Resist (2015) 21(5):527–36. 10.1089/mdr.2014.0215 26430941

[54] Danish Agriculture and Food Council. Statistics 2015 – Pig Meat. (2016). 48 p. Available from: http://www.lf.dk/tal-og-analyser/aarstatistikker/statistik-svin/statistik-svin-2015

[55] Korsgaard H Rosenquist H Agersø Y Human Risk Related to Spreading of Swine-MRSA from the Farm Environment (in Danish). Søborg, Denmark: DTU Food, Danish Technical University (2016).

[56] Statens Serum Institute. Increasing Incidence of MRSA Bacteria (in Danish). EPI-NEWS (2012). Available from: http://www.ssi.dk/aktuelt/nyheder/2012/2012_10_stigende%20forekomst%20af%20mrsa_041012.aspx

[57] de Boer E Zwartkruis-Nahuis JT Wit B Huijsdens XW de Neeling AJ Bosch T Prevalence of methicillin-resistant *Staphylococcus aureus* in meat. Int J Food Microbiol (2009) 134(1–2):52–6. 10.1016/j.ijfoodmicro.2008.12.007 19144432

[58] EFSA. Assessment of the public health significance of methicillin resistant *Staphylococcus aureus* (MRSA) in animals and foods. Scientific opinion of the panel on biological hazards on a request from the European Commission. EFSA J (2009) 993:1–73. 10.2903/j.efsa.2009.993

[59] Gilbert MJ Bos MEH Duim B Urlings BAP Heres L Wagenaar JA Livestock-associated MRSA ST398 carriage in pig slaughterhouse workers related to quantitative environmenal exposure. Occup Environ Med (2012) 69:472–8. 10.1136/oemed-2011-100069 22544853

[60] Statens Serum Institute. EPI-NEWS No. 24a. (2014). Available from: http://www.ssi.dk/English/News/EPI-NEWS/2014/No%2024a%20-%202014.aspx

[61] Larsen J Petersen A Sørum M Stegger M van Alphen L Valentiner-Branth P Methicillin-resistant *Staphylococcus aureus* CC398 is an increasing cause of disease in people with no livestock contact in Denmark, 1999 to 2011. Euro Surveill (2015) 20(37). 10.2807/1560-7917.ES.2015.20.37.30021 PMC4902279 26535590

[62] Van Cleef BAGL Broens EM Voss A Huijsdens XW Züchner L Van Benthem BHB High prevalence of nasal MRSA carriage in slaughterhouse workers in contact with live pigs in The Netherlands. Epidemiol Infect (2010) 138:756–63. 10.1017/S0950268810000245 20141647

[63] Normanno G Dambrosio A Lorusso V Samoilis G Di Taranto P Parisi A Methicillin-resistant *Staphylocoocus aureus* (MRSA) in slaughtered pigs and abattoir workers in Italy. Food Microbiol (2015) 51:51–6. 10.1016/j.fm.2015.04.007 26187827

[64] Jakobsen M Skovgaard CMB Jensen ML Wolf RT Rasmussen SR Det Nationale Institut for Kommuners og Regioners Analyse og Forskning (The Danish Institute for Local and Regional Government Research). Copenhagen, Denmark (2015). 64 p. Available from: https://www.kora.dk/english/

[65] Pinholt M Østergaard C Arpi1 M Bruun NE Schønheyder HC Gradel KO Incidence, clinical characteristics and 30-day mortality of enterococcal bacteraemia in Denmark 2006–2009: a population-based cohort study. Clin Microbiol Infect (2014) 20(2):145–51. 10.1111/1469-0691.12236 23647880

[66] ECDC/EMA. The Bacterial Challenge: Time to React. A Call to Narrow the Gap between Multidrug-Resistant Bacteria in the EU and the Development of New Antibacterial Agents. Technical Report. (2009). Available from: http://ecdc.europa.eu/en/publications/Publications/0909_TER_The_Bacterial_Challenge_Time_to_React.pdf

[67] ECDC. Point Prevalence Survey of Healthcare-Associated Infections and Antimicrobial Use in European Acute Care Hospitals. Stockholm, Sweden (2013). Available from: http://ecdc.europa.eu/en/publications/Publications/healthcare-associated-infections-antimicrobial-use-PPS.pdf

[68] Larsen J Clasen J Hansen JE Paulander W Petersen A Larsen AR Copresence of tet(K) and tet(M) in livestock-associated methicillin-resistant *Staphylococcus aureus* clonal complex 398 is associated with increased fitness during exposure to sublethal concentrations of tetracycline. Antimicrob Agents Chemother (2016) 60:4401–3. 10.1128/AAC.00426-16 27161637 PMC4914685

[69] Courvalin P. Predictable and unpredictable evolution of antibiotic resistance. J Intern Med (2008) 264(1):4–16. 10.1111/j.1365-2796.2008.01940.x 18397243

[70] WHO. Critically Important Antimicrobials for Human Medicine. 4th Revision (2016). 33 p. Available from: http://www.who.int/foodsafety/publications/antimicrobials-fourth/en/

